# SIRPα blockade improves the antitumor immunity of radiotherapy in colorectal cancer

**DOI:** 10.1038/s41420-023-01472-4

**Published:** 2023-06-09

**Authors:** Kai Ji, Yuhan Zhang, Shengpeng Jiang, Lin Sun, Baozhong Zhang, Dongzhi Hu, Jun Wang, Lujun Zhao, Ping Wang, Zhen Tao

**Affiliations:** 1 Department of Pain Relief, Tianjin Medical University Cancer Institute and Hospital, National Clinical Research Center of Cancer, Key Laboratory of Cancer Prevention and Therapy, Tianjin, and Tianjin’s Clinical Research Center for Cancer, Tianjin, P. R. China; 2 Department of Radiation Oncology, Tianjin Medical University Cancer Institute and Hospital, National Clinical Research Center of Cancer, Key Laboratory of Cancer Prevention and Therapy, Tianjin, and Tianjin’s Clinical Research Center for Cancer, Tianjin, P. R. China; 3 Department of Pathology, Tianjin Medical University Cancer Institute and Hospital, National Clinical Research Center of Cancer, Key Laboratory of Cancer Prevention and Therapy, Tianjin, and Tianjin’s Clinical Research Center for Cancer, Tianjin, P. R. China; 4 Department of Colorectal Cancer, Tianjin Medical University Cancer Institute and Hospital, National Clinical Research Center of Cancer, Key Laboratory of Cancer Prevention and Therapy, Tianjin, and Tianjin’s Clinical Research Center for Cancer, Tianjin, P. R. China; 5 Department of Oncology, Tongji Hospital, Tongji Medical College, Huazhong University of Science and Technology, Wuhan, Hubei P. R. China

**Keywords:** Tumour immunology, Radiotherapy

## Abstract

High-dose hypofractionated radiotherapy (HRT) is an important anticancer treatment modality that activates antitumor host immune responses. However, HRT for oligometastases of colorectal cancer (CRC) has shown frustrating results in the clinic. As part of immune evasion, myeloid cells express signal regulatory protein α (SIRPα) to inhibit phagocytosis by phagocytes in the tumor microenvironment (TME). We postulated that SIRPα blockade enhances HRT by alleviating the inhibitory action of SIRPα on phagocytes. We demonstrated that SIRPα on myeloid cells was upregulated in the TME after HRT. When SIRPα blockade was administered with HRT, we observed superior antitumor responses compared with anti-SIRPα or HRT alone. When anti-SIRPα was administered to local HRT, the TME could become a tumoricidal niche that was heavily infiltrated by activated CD8^+^ T cells, but with limited myeloid-derived suppressor cells and tumor-associated macrophages. While CD8^+^ T cells were required for the effectiveness of the anti-SIRPα + HRT combination. The triple therapy with anti-SIRPα + HRT + anti-PD-1 had superior antitumor responses compared with the combination of any two therapies and established a strong and long-lasting adaptive immunological memory. Collectively, SIRPα blockade provides a novel way to overcome HRT resistance in oligometastatic CRC patients. Our results herein provide a valuable cancer treatment strategy that has the potential to be translated into clinical practice.

## Introduction

Colorectal cancer (CRC) is the second highest reason of cancer mortalities worldwide [1]. The 5-year overall survival (OS) for metastatic CRC has historically been less than 15% [2]. As the current representative approach for cancer immunotherapy, immune checkpoint blockade (ICB), like anti-PD-1, is effective in a subset of metastatic CRC patients with DNA mismatch repair-deficient (dMMR)/microsatellite instability-high (MSI-H), but not in metastatic CRC patients with mismatch repair–proficient (pMMR)/microsatellite stable (MSS), which represents 95% of metastatic CRC cases [3–5]. The aggregation of myeloid cells in the tumor microenvironment (TME) is linked to poor prognosis and T-cell ICB resistance [6, 7]. Therefore, therapies targeting immune checkpoints on myeloid cells have potential value. As a critical innate immune checkpoint, signal-regulatory protein α (SIRPα) showed its expression on the plasma membrane of all myeloid cells, including monocytes, macrophages, granulocytes, myeloid dendritic cells, as well as microglia [8, 9]. Additionally, SIRPα expression has been observed in brain tissue [10], as well as on a subpopulation of CD8^+^ T cells throughout the course of chronic viral infections [11]. Its primary purpose is to engage with CD47, a self-recognition marker, to reduce professional phagocytes from engulfing self-cells, together with tumor cells [12, 13]. In several instances, an activating signal that identifies a target cell population for destruction will be necessary to suppress SIRPα-mediated inhibitory signaling [13]. Nevertheless, the action of single-agent SIRPα blockade is constrained because very few tumors have an endogenous activating signal that is potent enough.

Delivery of highly conformal, high-dose hypofractionated radiotherapy (HRT) has emerged as an effective local treatment for primary or oligometastatic CRC [14–16]. Aside from the well-characterized DNA damage-based mechanisms, HRT activates phagocytes by stimulating the translocation of calmodulin from the endoplasmic reticulum to the plasma membrane within tumor cells [17, 18]. However, the local failure rate of HRT for CRC oligometastases has been found to be unacceptably higher than for other primary cancers [15, 16]. An increase in myeloid cells in the TME after radiotherapy may be one of the causes of radioresistance [18]. Therefore, it is a possible future method for estimating the synergism of combining SIRPα blockade with HRT to boost the locoregional response of radioresistant histology such as CRC oligometastases.

Herein, we report that SIRPα is upregulated on myeloid cells in the TME after HRT. The combination of HRT and SIRPα blockade displays strong antitumor efficacy and modifies the TME that ultimately robustly activates the cytotoxicity of CD8^+^ T cells. Furthermore, we add PD-1 blockade to the combination therapy and find that this triple therapy not only significantly improves the local antitumor response but also induces long-term antitumor immunologic memory. Combined with these therapies, our results herein provide a valuable cancer treatment strategy that has the potential to be translated into clinical practice.

## Results

### SIRPα and/or CD47 are significantly overexpressed in CRC

**Fig. 1:**
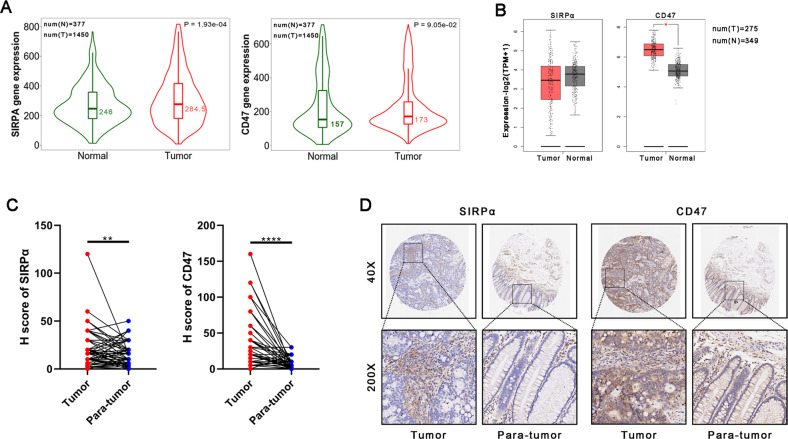
SIRPα and/or CD47 are significantly overexpressed in colorectal cancer. **A, B** Analyses of SIRPα and CD47 expressions in human colon cancer and normal counterparts from gene chip datasets or TCGA/GTEx are determined by TNM plot (A) or GEPIA2 (B). *P < 0.01. The Cancer Genome Atlas (TCGA); Genotype-Tissue Expression (GTEx). **C, D** Immunohistochemical (IHC) labeling of SIRPα and CD47 protein in CRC and adjacent healthy colon/rectal tissue controls (para-tumor tissue) in human tissues; total IHC score of SIRPα and CD47 in colorectal cancer and para-cancer tissues (*n* = 86) (**C**); Exemplary micrographs are displayed at their original magnification (200×) as indicated (**D**). Statistical differences were examined using the paired Student t test (C). **P < 0.01, ****P < 0.0001.

Based on past research, a high level of activation of SIRPα-CD47 signaling allows cancer cells to avoid immune detection and elimination by professional phagocytes [13, 19]. Thus, we evaluated SIRPα and CD47 expression in human colon cancer. When utilizing the gene chip databases in TNM plot [20] for analysis, SIRPα expression was higher in colon cancer compared with normal counterparts, while CD47 expression did not differ compared to normal counterparts (Fig. 1A). When evaluated using TCGA and GTEx datasets [21], we observed that CD47 expression was markedly higher in colon cancer contrasted with healthy counterparts, while SIRPα expression was not different (Fig. 1B). To further elucidate the expression of SIRPα-CD47 axis in CRC tissues, IHC testing was performed on a CRC tissue array that had 86 different cases of CRC as well as neighboring healthy controls. We found that compared to adjacent normal colon/rectal tissues, both SIRPα and CD47 expression were significantly upregulated in CRC (Fig. 1C, D). These findings suggested that it was potential to target the SIRPα-CD47 axis for CRC therapy.

### Increased SIRPα expression on myeloid cells in TME following HRT and combining SIRPα blockade with local HRT synergistically inhibit CRC growth in vivo

**Fig. 2:**
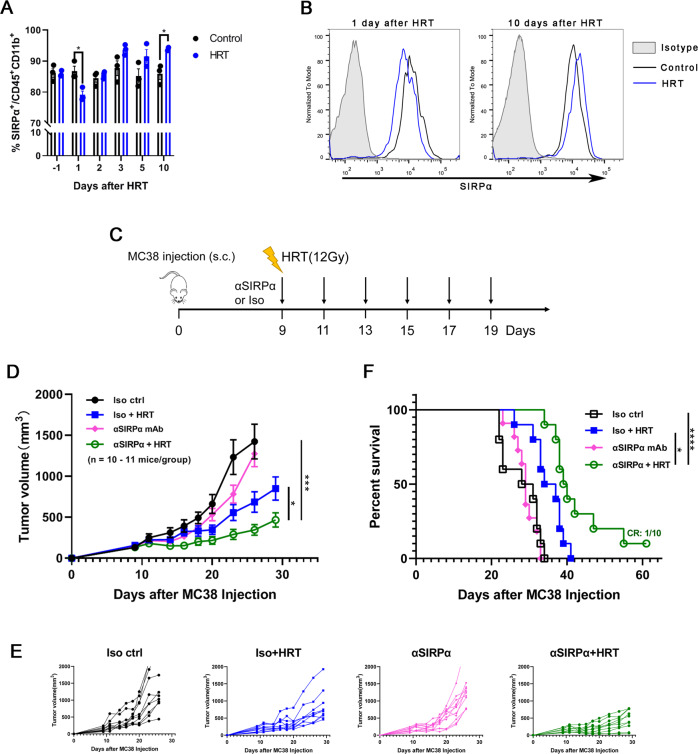
Increased SIRPα expression on myeloid cells in TME following HRT and SIRPα blockade combined with HRT synergistically inhibit colorectal cancer growth in vivo. **A, B** C57BL/6 mice were given through s.c. injection into the right flank with 1 × 10^6^ MC38 cells. Once tumor size achieved 100 mm^3^ (about 9 days after injection), a single 12 Gy dose of HRT was administered locally to mice. At 1, 2, 3, 5, and 10 days after HRT, SIRPα expression on myeloid cells was analyzed. **A** SIRPα expression on myeloid cells at different time points after HRT. **B** Representative data of twice experiments done with three mice per group are displayed. **C** 1 × 10^6^ MC38 cells were given into C57BL/6 mice, and 100 mm^3^ tumors were permitted to grow (around 9 days following injection). Subsequently, mice were randomly grouped (*n* = 10–11 per group), and given anti-SIRPα antibody (or isotype control), HRT, or combined, as depicted. Tumor growth curves of each treatment group are shown (**D, E**) with corresponding survival data (**F**). Experiments were repeated twice. Statistical variations were analyzed utilizing the unpaired Student t test (**A, D**) or log-rank test (**F**). *P < 0.05; ***P < 0.001; ****P < 0.0001.

In most cases, HRT seems effective with great local control of oligometastases, but metastases from CRC are an exception [15, 16]. One possible reason for this is that, despite the immunostimulating effects of HRT, the alteration of checkpoint levels in the TME may block the antitumor immunity by HRT. For exploring if HRT triggers SIRPα upregulation in TME, we removed the tumor tissue under flow cytometric testing of SIRPα expression on myeloid cells at different time points after performing HRT on MC38 tumors with 12 Gy. Compared to the identical cell subsets in control tumors, SIRPα expression levels on myeloid cells exhibited a decrease and thereafter a rise following HRT, and this difference was statistically significant on day 10 after HRT (Fig. 2A, B). In light of these data, SIRPα-mediated negative control of tumor-infiltrating phagocytes may be a significant host-mediated mechanism of tumor-associated acquired radioresistance.

We hypothesized that SIRPα blockade will improve HRT via diminishing its suppressive action on phagocytes. As depicted in Fig. 2C, MC38 tumor-bearing C57BL/6 mice were given either anti-SIRPα antibody, HRT (12 Gy), or both to study this concept. Anti–SIRPα alone had minimal impact on tumor proliferation, but HRT alone moderately delayed the progress of the tumor. However, the combination effectively inhibited MC38 tumor growth compared to anti-SIRPα (*P* = 0.005) and HRT alone (*P* = 0.034) (Fig. 2D, E). Additionally, survival among HRT (*P* = 0.0025) and combination-treated (*P* < 0.0001) mice was significantly higher than survival among controls, and one mouse obtained a complete response (CR) to combination therapy (Fig. 2F). To evaluate whether the combination of HRT with anti-SIRPα could result in an abscopal effect on the nonirradiated tumors, we implanted MC38 tumor cells in both flanks of mice, and only the primary tumors (right flank) were irradiated. Combination therapy slowed the growth rate of nonirradiated tumors compared with any single treatment (Supplementary Fig. 1). These results demonstrated that anti-SIRPα not only improves the effect of HRT on the primary tumor but also enhances the systemic abscopal effect.

### Combining SIRPα blockade with HRT reshapes the immunosuppressive TME

**Fig. 3:**
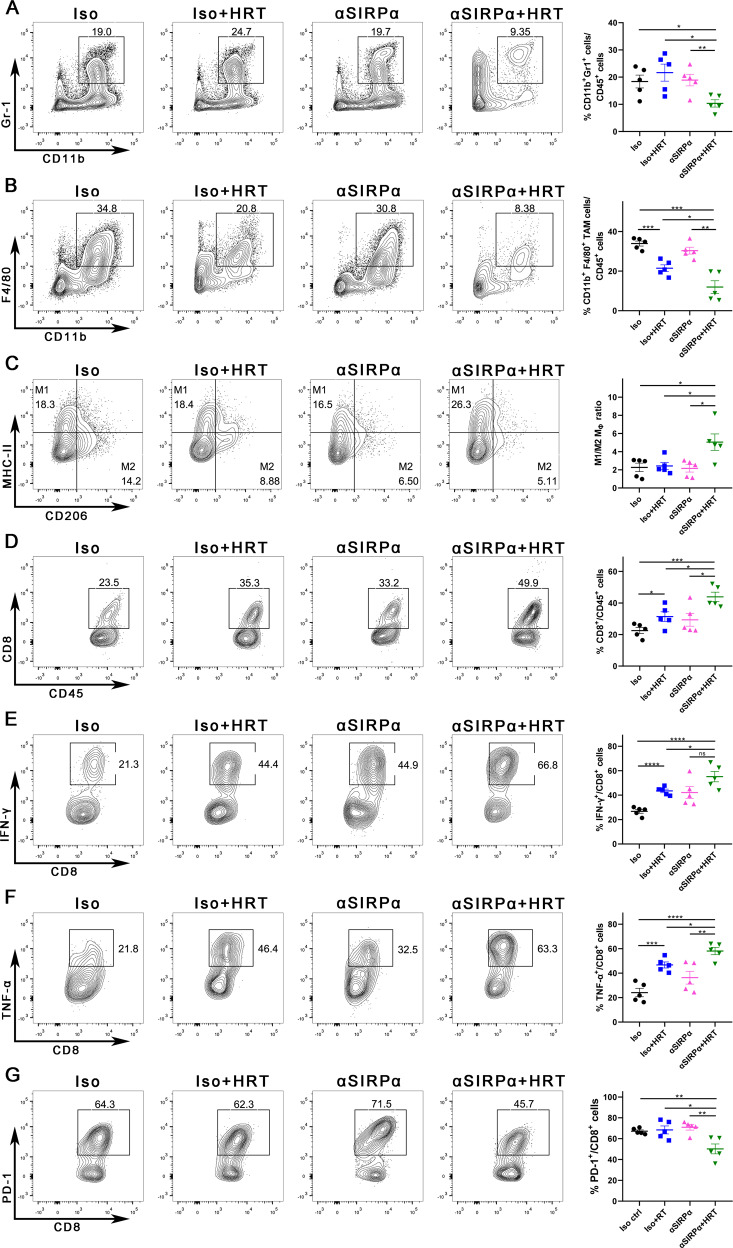
SIRPα blockade combined with HRT reshapes the immunosuppressive TME. C57BL/6 mice were injected s.c. with MC38 cells and treated with anti-SIRPα antibody (or isotype control), HRT, or both as in Fig. 2C. Ten days after HRT, immune cell populations in the TME were analyzed using flow cytometry. **A** Quantification of MDSCs (CD11b^+^Gr-1^+^) as a proportion of live CD45^+^ cells in the tumor. **B** Quantification of TAMs (CD11b^+^F4/80^+^) as a proportion of live CD45^+^ cells in the tumor. **C** M1 (F4/80^+^MHC-II^+^CD206^-^)/M2 (F4/80^+^MHC-II^-^CD206^+^) macrophage ratio in the tumor. **D** Quantification of CD8^+^ T cells as a proportion of live CD45^+^ cells in the tumor. **E, F** Quantification of IFN-γ^+^ (**E**) and TNF-α^+^ (**F**) cells as a proportion of CD8^+^ cells in the tumor. **G** Quantification of PD-1^+^ cells as a proportion of CD8^+^ cells in the tumor. All plots show a representative sample (left) and are expressed as a mean with 5 plotted replicates (right). Experiments were repeated twice. Statistical differences were assessed using the unpaired Student t test. *P < 0.05; **P < 0.01; ***P < 0.001; ****P < 0.0001.

As seen in the above results, anti-SIRPα alone had almost no effect on tumor control, but the addition of HRT significantly inhibited tumor growth compared to HRT. To understand the mechanism that underlies the exceptional tumoricidal ability of combined treatment, we conducted immunophenotyping of the TME in four groups of mice at 10 days after HRT. HRT alone slightly increased myeloid-derived suppressor cells (MDSCs, CD45^+^CD11b^+^Gr-1^+^) cell infiltration; however, the addition of anti-SIRPα reduced CD11b^+^Gr-1^+^ cell subsets dramatically among CD45^+^ cells in the TME (*P* = 0.0106 for anti-SIRPα + HRT vs. HRT, *P* = 0.0095 for anti-SIRPα + HRT vs. anti-SIRPα; Fig. 3A). HRT alone decreased the tumor-associated macrophages (TAMs, CD45^+^CD11b^+^F4/80^+^) phenotype among CD45^+^ cells, which was further decreased by adding anti-SIRPα (*P* = 0.0004 for HRT vs. Iso control, *P* = 0.0308 for anti-SIRPα + HRT vs. HRT; Fig. 3B). Whereas, anti-SIRPα alone did not affect both MDSC and TAM (Fig. 3A, B). For regulatory T cells (Tregs, CD4^+^CD25^+^Foxp3^+^) among CD45^+^CD4^+^ cells, neither HRT nor anti-SIRPα nor a combination of both showed differences from control (Supplementary Fig. 2A). Due to MDSCs, TAMs, and Tregs being 3 cell subsets that inhibit antitumor immunological response; moreover, can contribute to the growth of the tumor [22–24]. As a result, combination therapy helped eliminate the immunosuppressive cells from the TME.

**Fig. 4:**
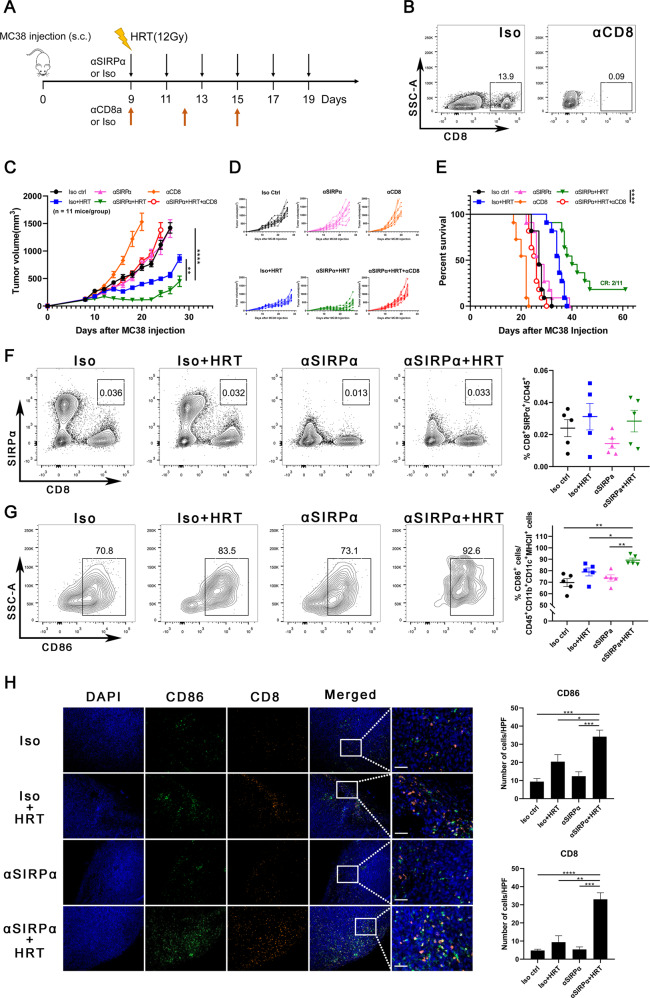
CD8^+^ T cells are essential for the antitumor effect of anti-SIRPα/HRT combination therapy. **A** MC38 tumors were again permitted to grow in mice. Around nine days after injection (tumor volume reached 100 mm^3^), mice were given anti-CD8 antibody, Iso control, HRT (12 Gy), anti-SIRPα antibody, or a triple therapy as depicted (*n* = 11 per group). **B** Depletion of intratumoral CD8^+^ cells by anti-CD8 antibody was assessed by flow cytometry. Tumor growth curves of every treatment group are depicted (**C, D**) with the matching survival results (**E**). **F-H** MC38 tumor-bearing mice were given anti-SIRPα antibody (or isotype control), HRT, or both as mentioned in Fig. 2C. Ten days following HRT, the lesions were resected. **(F**) Quantification of CD8^+^SIRPα^+^ cells as a proportion of live CD45^+^ cells in the tumor using flow cytometry. **G** Quantification of mature DCs (CD86^+^) as a proportion of CD11b^+^CD11c^+^MHC-II^+^ cells in the tumor using flow cytometry. **H** Multispectral immunofluorescence imaging reveals CD86^+^ and CD8^+^ cells infiltration in tumors. Scale bars: 50 μm. High-power field (HPF). All plots depict a typical sample (left) and are reported as the mean with five plotted replicates (right). Experiments were repeated twice. Statistical differences were assessed using the unpaired Student t test (**C, F**-**H**) or log-rank test (**E**). *P < 0.05; **P < 0.01; ***P < 0.001; ****P < 0.0001.

Macrophages in the TME can be roughly categorized into M1 and M2 types, which are believed to exert actions that block tumorigenesis and protumorigenic activities, respectively [25]. We discovered that the proportion of M1 macrophages to M2 macrophages in TME was significantly increased in the combination therapy mice contrasted with the mice in other groups (Fig. 3C). Additionally, we verified the serum levels of IL-12 and IL-10 by ELISA and the data were consistent with flow cytometry (Supplementary Fig. 3). Furthermore, HRT alone elevated CD8^+^ T-cell infiltration, while further addition of anti-SIRPα dramatically increased CD8^+^ T cells seen in the TME amongst CD45^+^ cells (*P* = 0.0408 for HRT vs. Iso control, *P* = 0.0184 for anti-SIRPα + HRT vs. HRT; Fig. 3D). Immunostaining of tumor tissues revealed that most CD8^+^ T cells in the HRT group were distributed at the tumor margins, whereas CD8^+^ T cells in the combined group were diffusely distributed throughout the tumor (Fig. 4H). For assessing if the combined medication elevated the activation and differentiation of CD8^+^ T cells, the TME was analyzed for CD8^+^ cells expressing Tc1 subtype markers, including IFN-γ and TNF-α. CD8^+^ T cells from tumors given combination therapy exhibited an elevated expression patterns of IFN-γ (*P* = 0.047 for anti-SIRPα + HRT vs. HRT, *P* < 0.0001 for anti-SIRPα + HRT vs. Iso control; Fig. 3E) and TNF-α (*P* = 0.019 for anti-SIRPα + HRT vs. HRT, *P* < 0.0001 for anti-SIRPα + HRT vs. Iso control; Fig. 3F). We also analyzed Immune checkpoint expression on CD8^+^ cells. Following combination therapy, CD8^+^ cells expressed less PD-1, TIM-3, and CD39 compared with those after anti-SIRPα or HRT alone (Fig. 3G, Supplementary Fig. 2B, C). Accordingly, the present findings demonstrated that combination therapy with anti-SIRPα and HRT promoted the activation of antitumor immunity within the TME.

### CD8^+^ T cells are essential for the antitumor effect of anti-SIRPα/HRT combination therapy

For further evaluation of the role of CD8^+^ T cells in the antitumor impact of the combined therapy, we blocked CD8^+^ cell infiltration into irradiated tumors through utilizing an anti-CD8 antibody starting on the HRT day (Fig. 4A). CD8 depletion was confirmed in tumors subjected to anti-CD8 antibody (Fig. 4B). Anti-CD8 antibody treatment resulted in tumors that were approximately the same size as those in the control group but significantly bigger than those treated with combined treatment (Fig. 4C, D). Two of 11 mice in the combination therapy group had a CR. Nevertheless, the depletion of CD8^+^ T-cell nullified the synergy between anti-SIRPα and HRT (Fig. 4E). Our results indicated that CD8^+^ T cells induced by combination treatment were important components for controlling CRC growth.

Considering that, in some cases, CD8^+^ T cells express SIRPα [11], to exclude the direct effect of SIRPα blockade on CD8^+^ T cells, we first examined the SIRPα expression on CD8^+^ T cells. We found that SIRPα was barely showed an expression on CD8^+^ T cells in MC38 tumors whether treated with anti-SIRPα, HRT, or both (Fig. 4F). Next, we examined mature dendritic cell (DC) markers CD45/CD11b/CD11c/MHC-II/CD86 in tumors to determine whether combination therapy affected phagocytes’ antigen-presenting ability. Not surprisingly, we found significantly more mature DCs in the combination therapy group (Fig. 4G) and more CD86^+^ cells infiltrating the tumors compared to anti-SIRPα and HRT alone (Fig. 4H). These findings indicate that CD8^+^ T cells act as a mediator for the immunological synergism between anti-SIRPα and HRT; in addition, the enhanced antigen presentation can explain CD8^+^ T-cell activation and differentiation.

### SIRPα blockade and HRT treatment leads antitumor response enhanced by PD-1 blockade

**Fig. 5:**
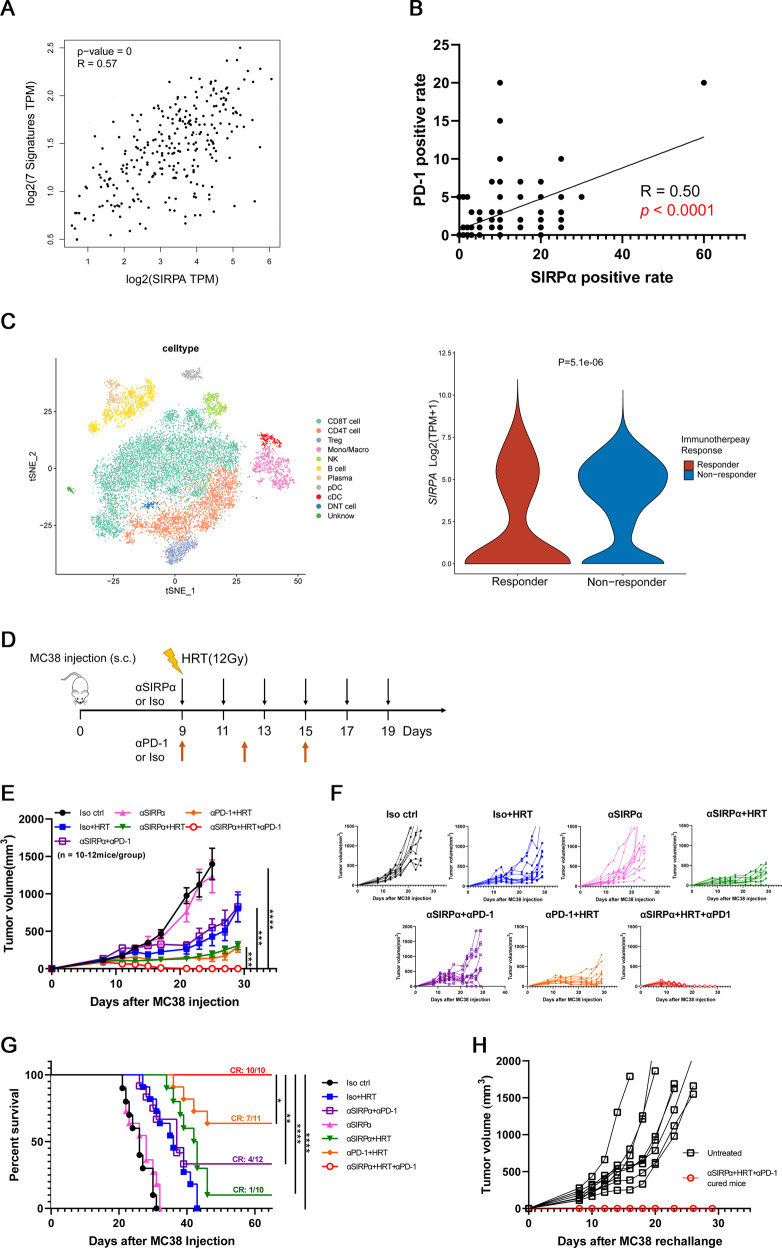
SIRPα blockade and HRT treatment leads antitumor response enhanced by PD-1 blockade. **A** Analyses of SIRPα gene and exhausted T cell signature genes expressions in human colon cancer from TCGA were determined by GEPIA2. Exhausted T cell signature genes included HAVCR2, TIGIT, LAG3, PDCD1, CXCL13, LAYN, and CD39. **B** IHC staining of SIRPα and PD-1 protein in colorectal cancer in human tissues; the correlation between SIRPα positivity rate and PD-1 positivity rate. **C** T-distributed stochastic neighbor embedding (t-SNE) plot of CD45^+^ immune cells from all samples, colored by identified cell clusters (left). The violin plot shows single-cell gene expression of SIRPα in immunotherapy response group and nonresponse group (right). Double negative T cell (DNT cell); Natural killer cell (NK). **D** MC38 tumors were afterward permitted to grow in mice. Around nine days after injection (tumor volume reached 100 mm^3^), mice were given anti-PD-1 antibody, Iso control, HRT (12 Gy), anti-SIRPα antibody, or a triple treatment as depicted (*n* = 10–12 per group). Tumor growth curves of each therapeutic group are depicted (**E, F**) with corresponding survival data (**G**). **H** Tumor growth curves of untreated naive (*n* = 8) or MC38 tumor-free mice given the αSIRPα + HRT + αPD-1 combination (*n* = 10) and rechallenged with 5 × 10^6^ MC38 cells 70 days after first tumor inoculation. Experiments were repeated twice. Statistical variations were analyzed utilizing the Pearson correlation analysis (**A, B**) or Wilcoxon test (**C**) or unpaired Student t test (**E**), or log-rank test (**G**). *P < 0.05; **P < 0.01; ***P < 0.001; ****P < 0.0001.

Myeloid cells are a main constituent of the TME [26]. Myeloid accumulation is linked to bad prognosis as well as resistance to T-cell ICB [6, 7, 22]. Given that SIRPα is predominantly expressed on myeloid cells, we first evaluated the correlation of SIRPα expression with the adaptive immune checkpoint PD-1. When evaluated using the TCGA dataset [21], we observed that SIRPα gene expression was linearly connected with exhausted T cell signature genes (Fig. 5A). To further elucidate the correlation, we detected the expression of SIRPα and PD-1 by IHC in a human CRC tissue array encompassing 94 CRC samples. We found that SIRPα positive rate was linearly correlated with PD-1 positive rate (Fig. 5B). Furthermore, by re-analysis of the public monocytic melanoma dataset GSE120575, we found that patients who did not respond to PD-1/CTLA4 inhibitors had higher SIRPα expression on monocyte/macrophage cells (Fig. 5C). This result implies that SIRPα overexpression maybe acts as a resistance mechanism to ICB therapy. Additionally, previous studies found that engaging both innate and adaptive checkpoints may induce durable antitumor response [27, 28]. Based on the above results, we hypothesized that using adaptive immune checkpoint blockade by neutralizing PD-1 could further improve the antitumor response of anti-SIRPα and HRT treatment. To test this hypothesis, MC38 tumor-bearing C57BL/6 mice were treated with anti-SIRPα antibody, HRT, anti-PD-1 antibody, or a combination of two or all three, as shown in Fig. 5D. Combination therapy with anti-SIRPα + HRT or anti-PD-1 + HRT or anti-SIRPα + anti-PD-1 significantly inhibited tumorous proliferation in C57BL/6 mice, while the CR was detected in just 10%–64% of mice. The addition of anti-PD1 to anti-SIRPα + HRT, on the other hand, greatly suppressed tumor progression in all mice and caused persistent CR in all mice (Fig. 5E, F, G). For evaluating immunity memory following triple treatment with anti-SIRPα + HRT + anti-PD-1, 10 full responders were re-challenged using 5 × 10^6^ (i.e., 5 fold the cell count of the first challenge) MC38 cells in the other flank 70 days following the first tumor inoculation, while 8 untreated naive mice served as controls. All recovered mice were resistant to a subsequent tumor challenge utilizing the exact cell line, demonstrating that triple therapy promotes memory immune responses (Fig. 5H, Supplementary Fig. 4).

### SIRPα expression is associated with prognosis of CRC patients

**Fig. 6:**
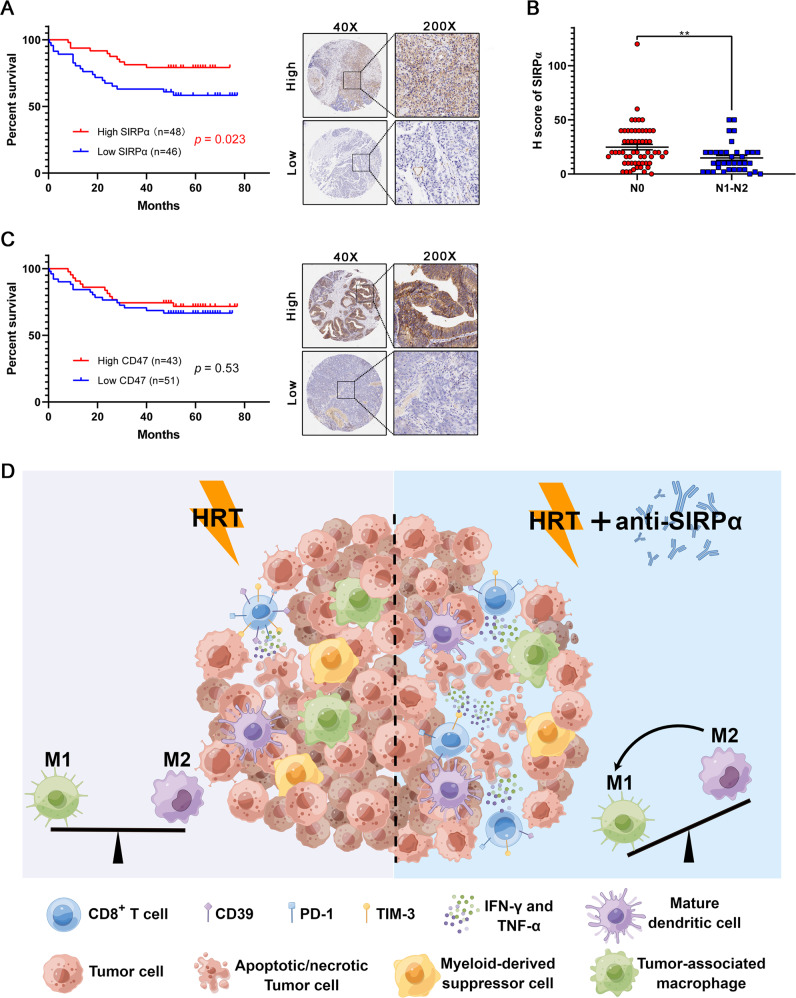
SIRPα expression is associated with prognosis of CRC patients. **A** Kaplan–Meier plot of OS for 94 patients having CRC, segmented according to SIRPα expression (left). Representative micrographs for IHC staining of SIRPα protein are shown (right). **B** Correlation of SIRPα expression with lymph node status. **C** Kaplan–Meier plot of OS for 94 patients having CRC, segmented according to CD47 expression (left). Representative micrographs for IHC staining of CD47 protein are shown (right). Statistical variations were analyzed utilizing the log-rank test (**A, C**) or unpaired Student t test (**B**). **P < 0.01. **D** The schematic summary of synergistic mechanism between SIRPα blockade and HRT. Left, HRT moderately activated antitumor immune response. It increased CD8^+^ T effector cells and reduced TAM infiltration. Right, the addition of anti-SIRPα antibody further reduced TAM infiltration within TME, while enabling M2 polarization to M1 and decreasing MDSCs infiltration. Furthermore, it stimulated the generation of TNF-α and IFN-γ and the recruitment of antigen-presenting cells and reduced T-cell exhaustion compared to HRT alone.

For evaluating the clinical value of SIRPα-CD47 axis expression in CRC patients, we used IHC to assess the protein expression profiles of SIRPα and CD47 in a tissue array of 94 human CRC samples. Kaplan–Meier survival analysis declared that CRC patients with high SIRPα expression showed longer OS (Fig. 6A), whereas CD47 expression showed no association with OS (Fig. 6C). Multivariate Cox hazards regression analysis detected vascular invasion (hazard ratio (HR) = 2.68, *P* = 0.009), lymph node metastasis (HR = 3.77, *P* = 0.004), and distant metastasis (HR = 5.66, *P* = 0.007) as possible distinct risk indicators for CRC patients (Supplementary table 1). Further analysis revealed that SIRPα expression correlated with free lymph node metastasis, but not with vascular invasion status or distant metastasis (Fig. 6B, Supplementary table 2). The current findings indicated that SIRPα expression may inhibit the occurrence of lymph node metastasis and thus prolong survival.

## Discussion

As a key immune checkpoint on phagocytes, SIRPα is capable of regulating a wide range of myeloid cell types. Inhibition of the SIRPα-CD47 axis often requires the simultaneous provision of an activation signal to mark the tumor cell for destruction. Conventional fractionated radiotherapy causes direct killing of tumor cells by means of irreversible DNA double-strand breaks manifesting as mitotic catastrophe and cellular apoptosis [29]. However, HRT could eliminate tumor cells through necrosis [30]. It is believed that necrosis triggers immune response, frequently characterized by the production of pro-inflammatory mediators as well as damage-associated molecular patterns that facilitate antigen presentation and T-cell priming for tumor cell killing [31]. However, HRT alone was less effective for CRC oligometastases compared to other primary tumors [15, 16]. Larger tumor size, lower biologically effective dose, and combined TP53 and KRAS mutations could partially explain the higher local failure rate [32]. Our study reveals that SIRPα enhances immunosuppression, confers HRT resistance in CRC, and thus ensures tumor progression. Here we demonstrate that HRT-induced immunity and enhanced SIRPα expression together provide a window of opportunity for the potent pharmacological effects of SIRPα inhibitors, reducing HRT resistance in CRC and increasing the rate of HRT-induced immune response. The present findings are consistent with a model in which the addition of anti-SIRPα to local HRT can transform the TME into a tumor-killing niche heavily populated by activated CD8^+^ T cells, but with limited MDSC and TAM (Fig. 6D).

Furthermore, our data suggest that the adaptive immunity, in particular CD8^+^ T cells, contributes to the management of CRC after combination therapy with anti-SIRPα and HRT. However, this observation cannot be accounted for by a direct effect of SIRPα blockade on T cell function, as SIRPα is barely expressed on CD8^+^ T cells. Recently, it was demonstrated that anti-CD47 antibody promoted antigen cross-presentation via DCs and boosted antigen-specific T cell activation in mice [27, 33]. Additionally, it has been reported that anti-CD47 antibody-mediated phagocytosis of tumor cells through macrophages can induce cytotoxic CD8^+^ antitumor T-cell reactions in mice [34]. In accord with these findings, we found that the addition of anti-SIRPα antibody augmented mature DCs as well as cells expressing CD86 costimulatory molecules infiltration in mice receiving local HRT. Coinciding with this, CD8^+^ T-cell reactions and Tc1 effector cell infiltration increase robustly. This indicates that anti-SIRPα and HRT have immunogenic synergy at the level of antigen presentation and T-cell priming. SIRPα, on the other hand, has a restricted expression pattern, while CD47 is ubiquitously expressed in all tissues and various types of cells. Therefore, the therapeutic window for anti-CD47 therapy is restricted since high dosages are required to overcome antigen sink. Moreover, unlike rodent models, humans and higher primates express SIRPγ, a SIRP homolog, on T cells [35]. Previous research found that blocking the binding of SIRPγ with CD47 inhibits human T-cell activation, proliferation, as well as migration across the endothelium, emphasizing the significance of targeting SIRPα in a selective manner [27].

Based on the Checkmate-142 study, Anti-PD-1 monotherapy improves OS in patients with dMMR/MSI-H metastatic CRC who had received prior chemotherapy [3]. Indirect comparisons suggest combination therapies using two types of T-cell ICB provide better efficacy than anti-PD-1 monotherapy, albeit with increased Grade 3 to 4 treatment-related adverse events [3, 36]. However, dMMR/MSI-H represent only approximately 5% of total newly diagnosed metastatic CRC cases [3]. While the remaining pMMR/MSS tumors are typically unresponsive to ICB therapy [4, 5]. Therefore, alternative therapies need to be identified to extend the benefits of immunotherapy beyond inflamed tumors. MYC oncogene, which plays a critical role in CRC, is known to promote CD47 and PD-L1 expression on cancer cells through binding to their corresponding promoters [37, 38]. Therefore, simultaneous blockade of innate and adaptive immune checkpoints together can be a promising therapy. Our study shows that triple treatment eliminated the main cancer and then led to develop the memory of the adaptive immune system that is strong and long-lasting in each mouse. Recently, it has been possible, through the development of bispecific antibodies, to target both the SIRPα-CD47 axis and the PD-1-PD-L1 axis in the TME, whereas concurrently lowering the antibodies’ off-target binding to normal cells [28, 39, 40]. Given that the bispecific treatment results in more favorable antitumor impacts contrasted to either SIRPα-CD47 axis or PD-1-PD-L1 axis blockade alone in mice tumor models, combined therapy with HRT and bispecific antibody warrants further investigation.

The expression of CD47 on tumor cells is currently considered to be an immune-tolerance mechanism, as it can inhibit professional phagocytes from contractile engulfment of tumor cells by interacting with SIRPα. Therefore, it is expected that this mechanism will result in a negative correlation between SIRPα-CD47 axis expression and survival as it was described, for example, for follicular lymphoma [41], diffuse large B-cell lymphoma [42], and esophageal squamous cell carcinoma [43]. Based on our findings, however, SIRPα expression was linked to a superior OS in patients with CRC, while CD47 expression exhibited no connection with survival. At present, it is still unclear why the upregulation of SIRPα is in certain cases (like CRC in the current research), regarded as a positive clinical effect. Notably, an earlier investigation also found a positive predictive role of SIRPα expression in CRC. In a study involving 269 primary CRCs, Sugimura-Nagata et al. reported high SIRPα tumor-associated immune cell counts are associated with better outcomes [44]. In addition, tumors with high levels of SIRPα were likely to display characteristics of the dMMR phenotype [44]. This may imply that SIRPα-high tumors have an active immune microenvironment resulting from high mutational burden and neoantigen load. Another explanation for the positive effect of SIRPα expression may be compensatory upregulation in a microenvironment where an active immune response threatens the tumor, similar to elevated SIRPα expression after HRT.

In summary, the combination of SIRPα blockade and HRT resulted in CRC regression, by resetting the immunosuppressing characteristics of the TME in a way that is reliant on CD8^+^ T-cell. Moreover, targeting both innate and adaptive checkpoints combined with HRT has extraordinary synergism, such as long-lasting systemic antitumor immunity. These data suggest that the combination with SIRPα blockade holds the promising potential to improve the prognosis of patients with oligometastatic CRC over HRT alone.

## Materials and methods

### Cell lines, mice, and reagents

Mouse CRC cell line MC38 was purchased from BMCR (http://www.cellresource.cn/). Cells were cultivated in RPMI-1640 enriched using 10% FBS, 10 mg/mL penicillin-streptomycin, and 0.1 mmol/L nonessential amino acids (all materials from Gibco) at 37 °C and 5% CO2. Six- to 8-week-old female C57BL/6 mice were acquired through SPF (Beijing) Biotechnology Co., Ltd. Food and water were available to all mice at all times under pathogen-free conditions. Anti-mouse SIRPα antibody (clone P84), anti-mouse CD8 antibody (clone YTS 169.4), anti-mouse PD-1 antibody (clone 29 F.1A12), and isotype controls were acquired through Bio X Cell.

### In vivo tumor growth experiments

Cellometer Mini Cell Counter (Nexcelom Biosience, Lawrence, MA) was used to count the cells after they had been cultured for less than two weeks. Female C57BL/6 mice aged 6 to 8 weeks were administrated through subcutaneous injection of 1 × 10^6^ MC38 cells at the right flank. Approximately 100 mm^3^ of tumor volume was reached after 8-9 days, after which treatments were assigned (n = 10-12 per group). On day 8 or 9, the C57BL/6 mice were treated with either anti-SIRPα antibody (200 mg per mouse) by intraperitoneal injection (i.p.), anti-PD-1 antibody (200 mg per mouse) by i.p., HRT of 12 Gy, or the combination. For the abscopal effect model, 1 × 10^6^ and 2 × 10^5^ MC38 cells were subcutaneously implanted into the right and left flank of mice, respectively. The HRT was delivered by 6 MV X-rays with an output dose rate of 500 MU/min, from a Varian 600CD (Varian Medical Systems, Palo Alto, CA) linear accelerator, prepared with a 120 leaf Millennium multileaf collimator (MLC). Both the gantry and collimation angles were 0 degree. Five separated radiation fields with the field size of 2 cm × 2 cm at the isocenter plane were formed using the MLC, so that 5 mice at most could be irradiated simultaneously. To ensure an adequate dose of radiotherapy, the tumor of each mouse was placed within 1 cm × 1 cm of the center of each radiation field, with the rest of the body placed as far outside the radiation field as possible. The mice were placed at the isocenter plane, on a 20 cm thickness solid water phantom, and a 1.5 cm thickness build-up was placed above the mice. Every two to three days, tumor diameters were recorded, and sizes were determined utilizing following formula: V = L × W^2^ × 0.5, where L and W represent the tumor’s long and short diameters, respectively. Tumor growth delays tests were conducted twice for verification. Mice were killed when they exhibited symptoms of illness or developed subcutaneous tumors that were approximately 1,500 mm^3^ in size. On days 0, 3, and 6 following radiation exposure, every mouse was injected i.p. with 200 mg of anti-CD8 antibody or rat IgG2b isotype control for the CD8^+^ T-cell depletion tests.

All animal experiments were performed in accordance to the animal experimental guidelines and approved by the Animal Ethics and Welfare Committee of Tianjin Medical University Cancer Institute and Hospital.

### Flow cytometry

Single-cell suspensions of tumors were prepared, and flow cytometry was carried out as mentioned earlier [45, 46]. Cells were stained with the following antibodies obtained from BioLegend: CD16/32 (clone 93, catalog No. 103132), Zombie UV™ Fixable Viability Kit (catalog No. 100752), CD45-PerCP/Cy5.5 (clone 30-F11, catalog No. 103132), CD45-APC/Cy7 (clone 30-F11, catalog No. 103116), CD11b-FITC (clone M1/70, catalog No. 101206), SIRPα-PE (clone BM8, catalog No. 123122), Gr-1-PerCP/Cy5.5 (clone RB6-8C5, catalog No. 108428), F4/80-AF647 (clone N418, catalog No. 117318),CD11c-PE/Cy7 (clone N418, catalog No. 117318), I-A/I-E-AF700 (clone M5/114.15.2, catalog No. 107622), CD86-BV421 (clone GL-1, catalog No. 105032), CD206-BV605 (clone C068C2, catalog No. 141721), CD8a-AF700 (clone 53-6.7, catalog No. 100730), CD8-BV510 (clone 53-6.7, catalog No. 100752), TNF-α-BV421 (clone MP6-XT22, catalog No. 506328), IFN-γ-PE (clone XMG1.2, catalog No. 505808), CD39-PE/Cy7 (clone Duha59, catalog No. 143806), Tim-3-APC/Fire™ 750 (clone RMT3-23, catalog No. 119738), PD-1-BV605 (clone 29 F.1A12, catalog No. 135220). The next antibodies were obtained through eBioscience: Mouse Regulatory T Cell Staining Kit #2 (clone FJK-16s, catalog No. 88-8118-40). FlowJo program (Tree Star Inc.) was utilized to examine the findings that were acquired through a BD LSRFortessa Flow Cytometer.

### Cytokine enzyme-linked immunosorbent assays (ELISA)

Quantification of serum cytokines (IL12/23p40 and IL10) was performed using ELISA Kit (DAKEWE, 1211232, 1211002) according to the manufacturer’s protocol. The plates were read at 450 nm within 10 minutes.

### Clinical samples and Immunohistochemical staining

A total of 94 CRC patients (86 had adjacent normal tissues) had paraffin tissue slices (HColA180Su21) acquired through Shanghai Outdo Biotech (National Human Genetic Resources Sharing Service Platform with code No. 2005DKA21300, Shanghai, China). CRC samples and controls were stained using immunohistochemical staining (IHC) in accordance with the recommended practices (Cell Signaling Technology). In brief, the slides were first incubated with primary antibodies: anti-SIRPα (1:500 dilution, Abcam, ab53721) or anti-CD47 (1:10000 dilution, Abcam, ab218810), followed by goat anti-rabbit secondary antibodies conjugated with horseradish peroxidase. A 2-Solution DAB Kit (Invitrogen) was used to visualize antibody binding. Independent scoring of the IHC results was performed by two expert pathologists who were blinded to the trial information. In the event of inconsistent scoring, the pathologists consulted with each other and re-reviewed the slides. Each tissue specimen was evaluated using a traditional H score for indicators, by the sum of relative intensity (0: negative; 1: weak; 2: moderate; 3: strong) of specific staining multiplied by the percentage of positive cells. Supplementary table 2 shows the clinical features of the patients (from whom tissues were obtained). For survival analyses, patient OS were categorized based on the median value of the scoring results and were denoted as Kaplan–Meier plots and examined for significance utilizing log-rank tests.

### Multiplexed immunofluorescence staining

To evaluate the density of the CD8^+^ and CD86^+^ cell composition of the TME and their relative spatial positioning in tumors, the PANO 4-plex IHC kit (catalog No. 10079100100, Panovue) was utilized for multiplex staining of tissue that was formalin-fixed and then embedded in paraffin. Following sequential application of CD8 (catalog No. 98941, CST) and CD86 (catalog No. 19589, CST) antibodies, horseradish peroxidase-conjugated secondary antibodies were incubated and tyramide signal amplification was performed. Following each tyramide signal amplification operation, the slides were microwave-heated. The DAPI stain was applied after all antigens were labeled. Multispectral images from the stained slides were acquired at 10× and 40× magnification using Zeiss Axio Imager Z2 multispectral microscope within the same exposure time. Each slide was scanned in five random areas without necrosis or damage.

### Bioinformatic analysis

We compared SIRPα and CD47 mRNA expressions of colon cancer and normal tissues by submitting a query to TNM plot [20] with selection of Gene chip data. As well, we obtained RNA-seq data from the Cancer Genome Atlas (TCGA) and the GTEx (Genotype-Tissue Expression) database using the GEPIA2 [21] tool, set to a *P*-value cutoff = 0.01, log2FC (fold change) cutoff = 1, and “matched TCGA normal and GTEx data”.

To analyze the correlation between SIRPα and exhausted T cell signature, RNA-seq data were obtained from the TCGA dataset using the GEPIA2 [21] tool. The T-cell-exhaustion signature gene set included HAVCE2, TIGIT, LAG3, PDCD1, CXCL13, LAYN, and CD39.

Public single-cell melanoma dataset GSE120575 was utilized to examine the connection between SIRPα expression and ICB resistance. The scRNA-seq analysis was carried out employing Seurat (V4.0.4). For quality control, the data was filtered to incorporate only genes that were expressed in a minimum of 5 cells and cells that expressed a minimum of 200 genes, with a maximum of 8000 genes and a minimum of 40,000 unique molecular identifier (UMI) counts and less than 15% of mitochondrial gene expression. Harmony (V0.1.0) was performed for removing the batch effects. The monocyte/macrophage cell type was defined through the expression of following marker genes: FCN1, VCAN, CD14, CD33, and CSF3R, as described in the original article [47].

### Statistical analysis

All findings were recovered from more than two separate experiments. Data were analyzed utilizing Prism 8.0 software (GraphPad Software). Results are denoted as the mean ± standard error of the mean (SEM) for all figure panels unless otherwise indicated. The *P* values were evaluated employing the independent Student *t*-test, paired Student *t*-test, Wilcoxon test, or the Pearson correlation test. Log-rank univariate analysis and Cox regression multivariate analysis were used to identify factors related to prognosis. Survival curves were displayed utilizing the Kaplan–Meier method and compared utilizing a log-rank test. Statistical significance was set to be a *P* value ≤ 0.05 in 2-tailed tests.

## Supplementary information

Supplementary figure legends

Supplementary Figure 1. SIRPα blockade combined with HRT synergistically inhibit both irradiated and abscopal tumors growth in vivo.

Supplementary Figure 2. Flow cytometric characterization of intratumoral Tregs and CD8+ cells.

Supplementary Figure 3.

Supplementary Figure 4.

Tumor volume of mice.

Supplementary Table 1. Log-rank test and multivariate cox regression of prognostic factors for survival of patients with colorectal cancer.

Supplementary Table 2. Clinicopathologic characteristics of SIRPα and CD47 expression in colorectal cancer patients.

## Data Availability

The data in this study are available within the article and its supplementary data files. Any other data can be obtained from the corresponding author on reasonable request.
