# Supplementary material for: SIRPα blockade improves the antitumor immunity of radiotherapy in colorectal cancer
Source: Cell Death Discov. 2023 Jun 9;9:180. doi: 10.1038/s41420-023-01472-4 (PMC10250547; doi:10.1038/s41420-023-01472-4)
Supplement: Supplementary file 1 — Supplementary figure legends [file 41420_2023_1472_MOESM1_ESM.docx]

**Supplementary Figure 1. SIRPα blockade combined with HRT synergistically inhibit both irradiated and abscopal tumors growth in vivo.** (A) Schematic diagram in mice implanted with bilateral MC38 tumors on flank subcutaneous regions and treated with HRT to right flank tumors, anti-SIRPα antibody, or combined (n = 8 per group). (B) Tumor growth curves of each treatment group are shown. Statistical variations were analyzed utilizing the unpaired Student t test. * *P* < 0.05; ** *P* < 0.01; **** *P*<0.0001.

**Supplementary Figure 2. Flow cytometric characterization of intratumoral Tregs and CD8^+^ cells.** C57BL/6 mice were treated as described in Figure 3. (A) Quantification of Tregs (CD25^+^Foxp3^+^) as a proportion of CD4^+^ cells in the tumor. (B) Quantification of TIM-3^+^ cells as a proportion of CD8^+^ cells in the tumor. (C) Quantification of CD39^+^ cells as a proportion of CD8^+^ cells in the tumor. All plots show a representative sample (left) and are expressed as a mean with 5 plotted replicates (right). Experiments were repeated twice. Statistical differences were assessed using the unpaired Student t test. ** *P* < 0.01.

**Supplementary Figure 3**. C57BL/6 mice were injected s.c. with MC38 cells and treated with anti-SIRPα antibody (or isotype control), HRT, or both as in Figure 2C. Ten days after HRT, ELISA was used to measure IL12/23P40 (A), and IL10 (B) in the serum of mice in various treatment groups. All data are expressed as a mean with 5 plotted replicates. Experiments were repeated twice. Statistical differences were assessed using the unpaired Student t test. ** P < 0.01; *** P<0.001; **** P<0.0001.

**Supplementary Figure 4.** Survival of untreated naive (n = 8) or MC38 tumor-free mice treated with the αSIRPα+HRT+αPD-1 combination (n = 10) and rechallenged with 5 × 10^6^ MC38 cells 70 days after first tumor inoculation.
