# Supplementary figures and images for: SIRPα blockade improves the antitumor immunity of radiotherapy in colorectal cancer
Source: Cell Death Discov. 2023 Jun 9;9:180. doi: 10.1038/s41420-023-01472-4 (PMC10250547; doi:10.1038/s41420-023-01472-4)

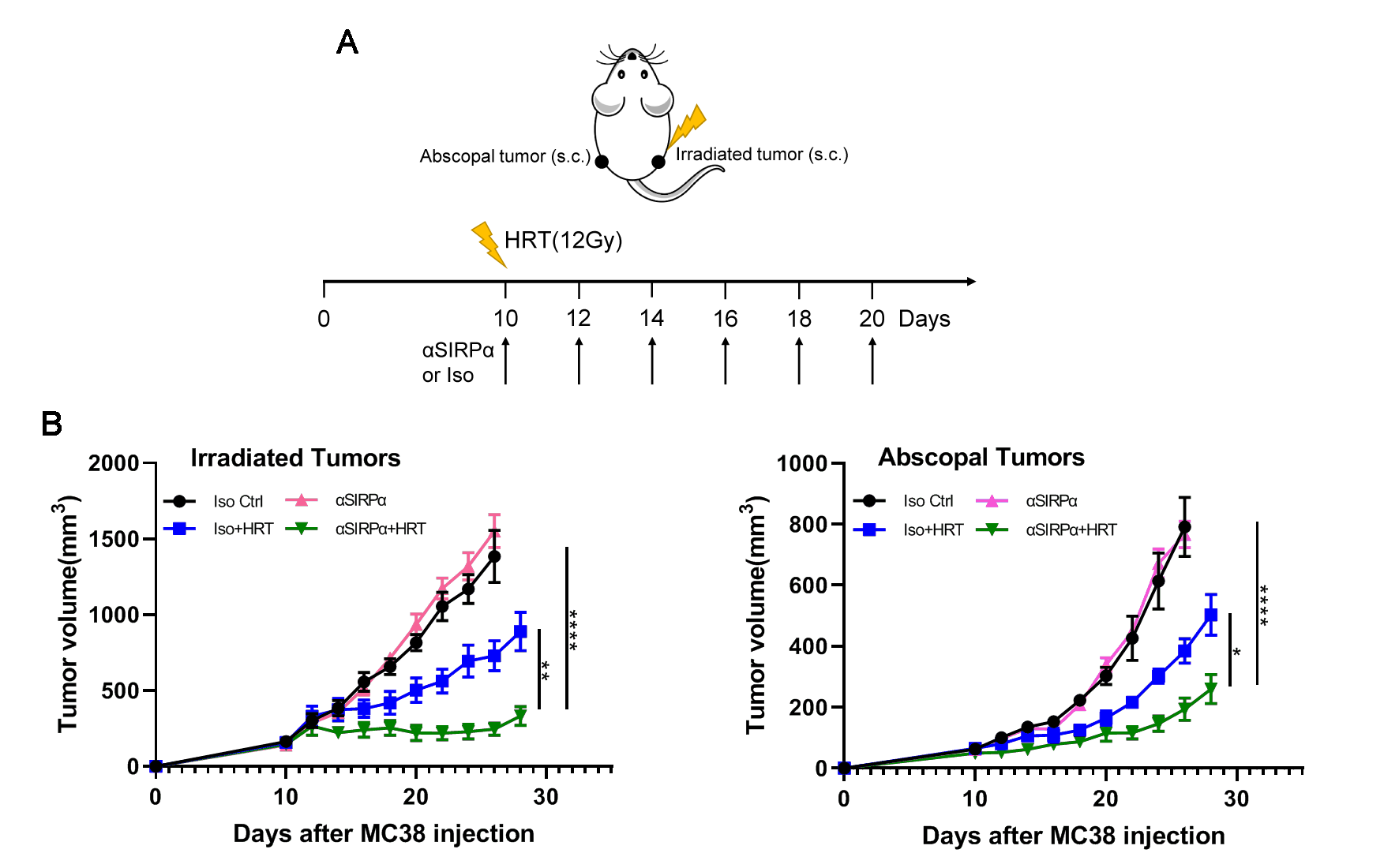

Supplement: Supplementary file 2 — Supplementary Figure 1. SIRPα blockade combined with HRT synergistically inhibit both irradiated and abscopal tumors growth in vivo. [file 41420_2023_1472_MOESM2_ESM.tif]

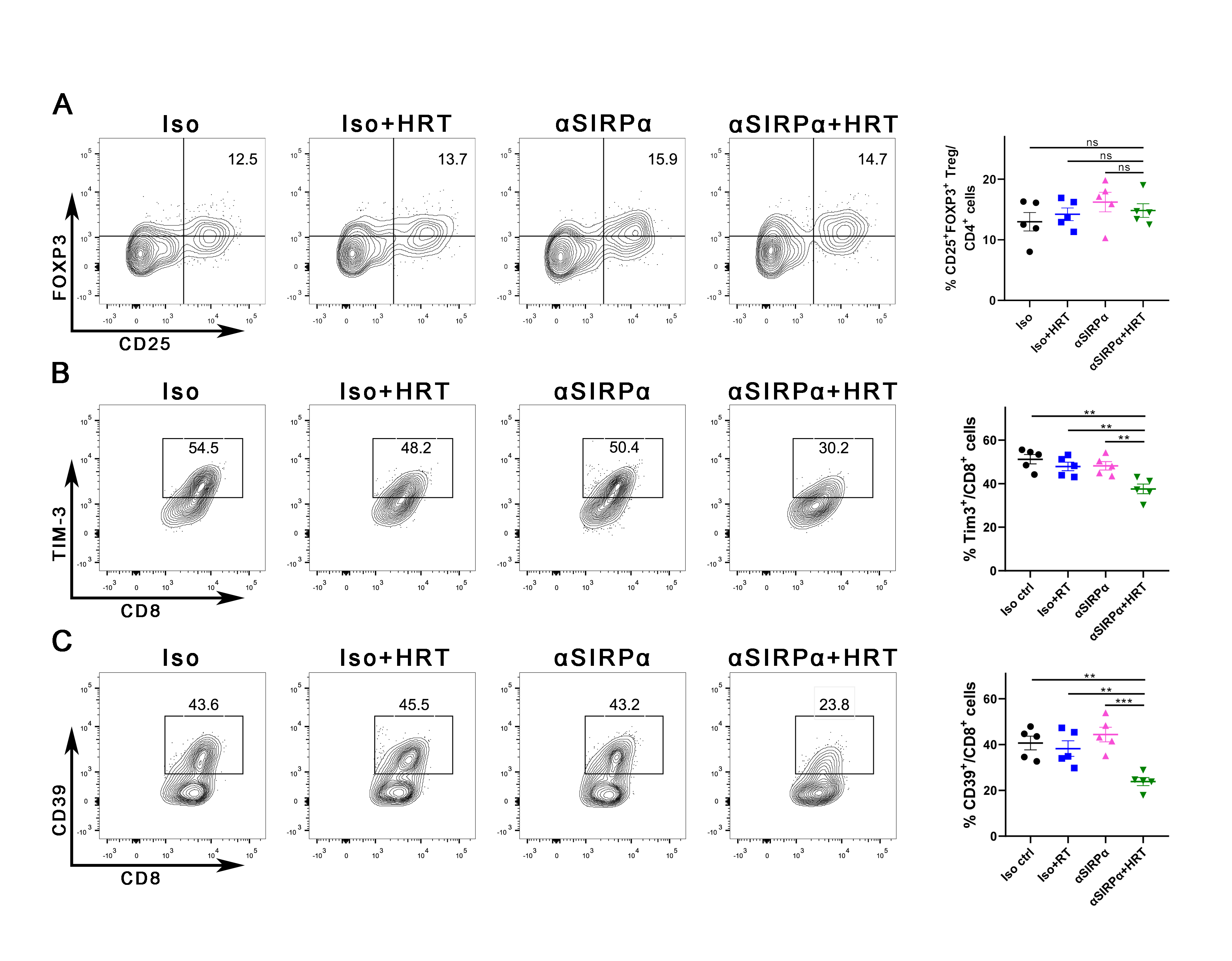

Supplement: Supplementary file 3 — Supplementary Figure 2. Flow cytometric characterization of intratumoral Tregs and CD8+ cells. [file 41420_2023_1472_MOESM3_ESM.tif]

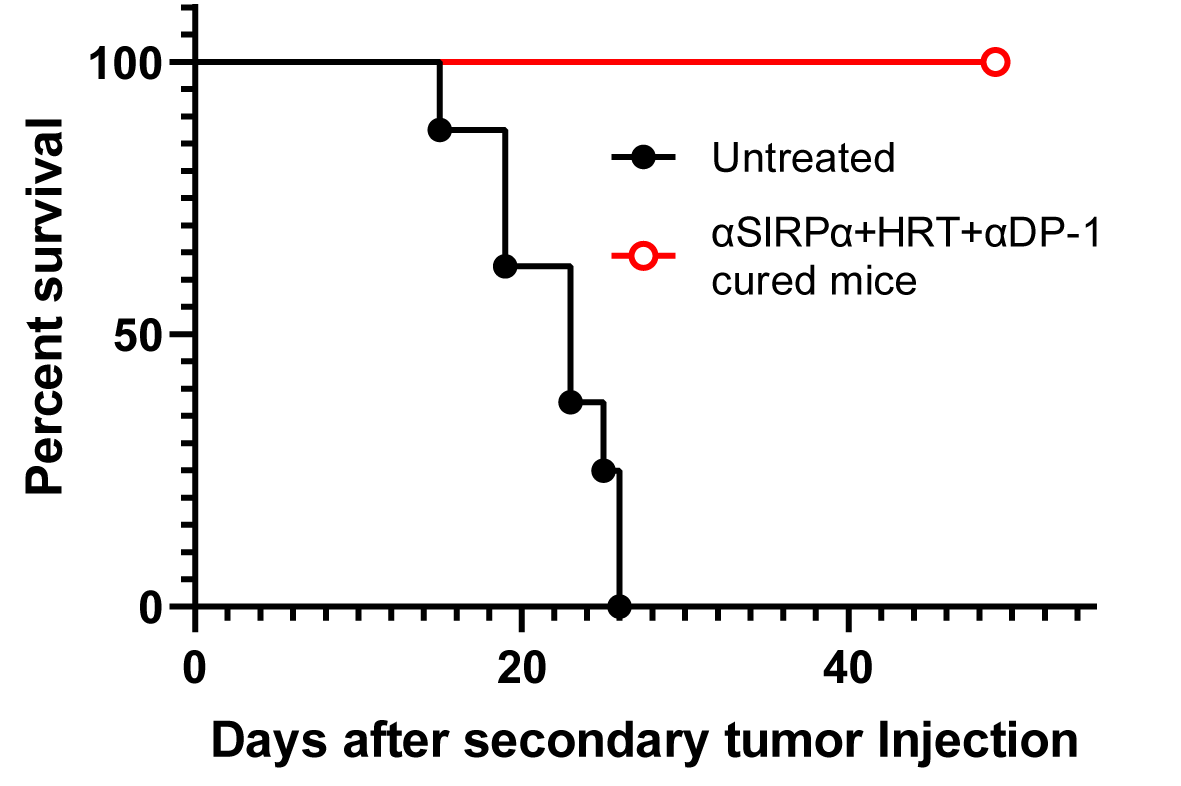

Supplement: Supplementary file 5 — Supplementary Figure 4. [file 41420_2023_1472_MOESM5_ESM.tif]
