# Supplementary material for: SIRPα blockade improves the antitumor immunity of radiotherapy in colorectal cancer
Source: Cell Death Discov. 2023 Jun 9;9:180. doi: 10.1038/s41420-023-01472-4 (PMC10250547; doi:10.1038/s41420-023-01472-4)
Supplement: Supplementary file 7 — Supplementary Table 1. Log-rank test and multivariate cox regression of prognostic factors for survival of patients with colorectal cancer. [file 41420_2023_1472_MOESM7_ESM.docx]

**Supplementary Table 1. Log-rank test and multivariate cox regression of prognostic factors for survival of patients with colorectal cancer.**

| **Characteristics** | **Univariate analysis** | | **Multivariate analysis** | |
| --- | --- | --- | --- | --- |
|  | **Mean OS (month)** | ***P* value^a^** | **HR value** | ***P* value^a^** |
| Age |  | 0.270 |  |  |
| ≤65 | 54.0 |  |  |  |
| ＞65 | 62.1 |  |  |  |
| Gender |  | 0.696 |  |  |
| Male | 59.4 |  |  |  |
| Female | 56.3 |  |  |  |
| Tumor type |  | 0.928 |  |  |
| Protuberant | 60.5 |  |  |  |
| Infiltrative | 54.0 |  |  |  |
| Ulcerative | 57.2 |  |  |  |
| Adenocarcinoma Pathological grade |  | 0.003 |  |  |
| II | 65.8 |  |  |  |
| III | 47.0 |  |  |  |
| Tumor size |  | 0.072 |  |  |
| <5 cm | 51.6 |  |  |  |
| ≥5 cm | 63.9 |  |  |  |
| Vascular invasion |  | 0.001 |  | 0.009 |
| No | 65.0 |  | Reference |  |
| Yes | 46.8 |  | 2.682 |  |
| Nerve invasion |  | 0.032 |  |  |
| No | 62.6 |  |  |  |
| Yes | 41.4 |  |  |  |
| T stage |  | 0.054 |  |  |
| T2 |  |  |  |  |
| T3/T4 |  |  |  |  |
| Lymph node metastasis^b^ |  | 0.000 |  | 0.004 |
| No | 66.4 |  | Reference |  |
| Yes | 45.3 |  | 3.768 |  |
| Distant metastasis |  | 0.037 |  | 0.007 |
| No | 60.2 |  | Reference |  |
| Yes | 25.0 |  | 5.655 |  |
| TNM staging |  | 0.001 |  |  |
| I/II | 66.1 |  |  |  |
| III/IV | 47.0 |  |  |  |
| CD8 expression |  | 0.185 |  |  |
| Low | 55.0 |  |  |  |
| High | 62.2 |  |  |  |
| PDL1 expression |  | 0.403 |  |  |
| Low | 57.0 |  |  |  |
| High | 60.0 |  |  |  |
| PD-1 expression |  | 0.012 |  |  |
| Low | 51.1 |  |  |  |
| High | 59.3 |  |  |  |
| SIRPα expression |  | 0.023 |  |  |
| Low | 51.9 |  |  |  |
| High | 63.2 |  |  |  |
| CD47 expression |  | 0.654 |  |  |
| Low | 55.6 |  |  |  |
| High | 60.7 |  |  |  |

^a^ *P* values were derived using parameter test to compare values for the two or three parameters in each category.

^b^ The tumor stage, lymph node status, and metastasis were classified according to the international system for staging CRC cancer.^1^

**Reference:**

1. Weiser MR. AJCC 8th Edition: Colorectal Cancer. Ann Surg Oncol 2018;25:1454-5.
