# Supplementary material for: SIRPα blockade improves the antitumor immunity of radiotherapy in colorectal cancer
Source: Cell Death Discov. 2023 Jun 9;9:180. doi: 10.1038/s41420-023-01472-4 (PMC10250547; doi:10.1038/s41420-023-01472-4)
Supplement: Supplementary file 8 — Supplementary Table 2. Clinicopathologic characteristics of SIRPα and CD47 expression in colorectal cancer patients. [file 41420_2023_1472_MOESM8_ESM.docx]

**Supplementary Table 2. Clinicopathologic characteristics of SIRPα and CD47 expression in colorectal cancer patients.**

| **Characteristics** | **Cases** | | **IHC score of SIRPα** | | |  | | **IHC score of CD47** | | | |
| --- | --- | --- | --- | --- | --- | --- | --- | --- | --- | --- | --- |
|  |  | **Mean±s.d.** | | ***P* value^a^** |  | | **Mean±s.d.** | | ***P* value^a^** | |  |
| Age (years)  *≤65*  *＞65* | 47  46 | | 21.4 ± 21.2  20.0 ± 14.1 | 0.691 | |  | | 32.0 ± 37.8  36.1 ± 32.2 | | 0.579 | |
| Gender  *Male*  *Female* | 46  48 | | 21.7 ± 20.8  20.4 ± 15.4 | 0.743 | |  | | 31.7 ± 35.8  35.9 ± 34.2 | | 0.562 | |
| Tumor type  *Protuberant*  *Infiltrative*  *Ulcerative* | 36  4  54 | | 20.4 ± 14.8  13.0 ± 8.7  22.0 ± 20.5 | 0.612 | |  | | 26.8 ± 25.0  53.8 ± 56.2  37.1 ± 38.3 | | 0.198 | |
| Pathological grade  *II*  *III* | 58  36 | | 23.5 ± 19.8  17.0 ± 14.4 | 0.090 | |  | | 30.4 ± 30.6  39.4 ± 40.7 | | 0.225 | |
| Tumor size  *<5 cm*  *≥5 cm* | 35  58 | | 22.1 ± 15.0  20.6 ± 19.9 | 0.690 | |  | | 31.0 ± 31.8  35.9 ± 37.0 | | 0.520 | |
| Vascular invasion  *No*  *Yes* | 62  31 | | 22.1 ± 14.4  19.2 ± 24.2 | 0.476 | |  | | 31.9 ± 33.0  38.2 ± 39.0 | | 0.417 | |
| Nerve invasion  *No*  *Yes* | 76  17 | | 20.3 ± 15.2  24.8 ± 28.4 | 0.358 | |  | | 34.3 ± 35.8  32.9 ± 32.1 | | 0.888 | |
| T stage  *T2*  *T3/T4* | 9  85 | | 18.9 ± 13.0  21.2 ± 18.6 | 0.713 | |  | | 21.1 ± 20.3  35.2 ± 35.9 | | 0.250 | |
| Lymph node status^b^  *N_0_*  *N_1-3_* | 58  36 | | 24.9 ± 19.7  14.8 ± 13.3 | 0.008 | |  | | 34.4 ± 36.9  33.1 ± 31.8 | | 0.857 | |
| Distant metastasis  *No*  *Yes* | 89  5 | | 21.0 ± 18.1  20.8 ± 19.6 | 0.978 | |  | | 31.7 ± 32.2  72.0 ± 59.3 | | 0.011 | |
| TNM stage  *I/II*  *III/IV* | 56  38 | | 25.1 ± 19.9  15.0 ± 13.3 | 0.007 | |  | | 31.3 ± 32.8  37.6 ± 37.8 | | 0.394 | |
| CD8 expression  *Low*  *High* | 48  46 | | 18.6 ± 15.3  23.5 ± 20.5 | 0.192 | |  | | 33.3 ± 38.5  34.5 ± 31.0 | | 0.877 | |
| PDL1 expression  *Low*  *High* | 36  57 | | 17.8 ± 13.5  23.4 ± 20.3 | 0.145 | |  | | 35.3 ± 38.2  33.4 ± 33.0 | | 0.805 | |
| PD1 expression  *Low*  *High* | 44  50 | | 12.9 ± 12.1  28.2 ± 19.6 | 0.000 | |  | | 38.2 ± 42.6  30.1 ± 26.1 | | 0.279 | |
| SIRPα expression  *Low*  *High* | 46  48 | |  |  | |  | | 35.5 ± 37.0  32.3 ± 33.0 | | 0.654 | |
| CD47 expression  *Low*  *High* | 51  43 | | 19.2 ±14.6  23.2 ± 21.5 | 0.285 | |  | |  | |  | |

^a^ *P* values were derived using parameter test to compare values for the two or three parameters in each category.

^b^ The tumor stage, lymph node status, and metastasis were classified according to the international system for staging CRC cancer.^1^

**Reference:**

1. Weiser MR. AJCC 8th Edition: Colorectal Cancer. Ann Surg Oncol 2018;25:1454-5.
